# Associations of latent patterns of parent‒child communication with communication quality and mental health outcomes among Chinese left-behind children

**DOI:** 10.1186/s12889-024-17793-7

**Published:** 2024-01-31

**Authors:** Qian-Wen Xie, Roujia Chen, Kexin Wang, Jingjing Lu, Feng Wang, Xudong Zhou

**Affiliations:** 1 Department of Social Welfare and Risk Management, School of Public Affairs, Zhejiang University, 866 Yuhangtang Rd, Hangzhou, 310058 Zhejiang China; 2 Research Center for Common Prosperity, Future Regional Development Laboratory, Innovation Center of Yangtze River Delta, Zhejiang University, Jiaxing, 314100 Zhejiang China; 3 Center of Social Welfare and Governance, Zhejiang University, 866 Yuhangtang Rd, Hangzhou, 310058 Zhejiang China; 4 School of Public Health, LKS Faculty of Medicine, The University of Hong Kong, Hong Kong SAR, China; 5 College of Media and International Culture, Zhejiang University, 866 Yuhangtang Rd, Hangzhou, 310058 Zhejiang China; 6 The Institute of Social Medicine, School of Medicine, Zhejiang University, 866 Yuhangtang Rd, Hangzhou, 310058 Zhejiang China; 7 Department of Health Policy and Management, School of Public Health, Hangzhou Normal University, 2318 Yuhangtang Rd, Hangzhou, Zhejiang China; 8 Hangzhou International Urbanology Research Center & Center for Urban Governance Studies, Hangzhou, China; 9 The Second Affiliated Hospital, School of Medicine, Zhejiang University, 88 Jiefang Road, Hangzhou, 310009 Zhejiang China

**Keywords:** Parent‒child communication, Mental health, Labor migration, Left-behind children, Health inequity, China

## Abstract

**Background:**

Parent‒child communication in migrant families is essential to family bonds and the mental health of left-behind children (LBC). Little is known about the different patterns of communication between migrant parents and LBC and associated communication quality and mental health outcomes.

**Methods:**

A sample of 2,183 Chinese children (mean age = 12.95 ± 1.29 years) from Anhui province, including LBC whose parents had both migrated (*n* = 1,025) and children whose parents had never migrated (never-LBC, *n* = 1,158), was analyzed. With the LBC sample, latent class analysis was applied to identify the patterns of parent‒child communication. Multinomial logistic regression analysis was conducted to assess the associations between the sociodemographic variables and class membership of LBC. Analysis of covariance and chi-square tests were used to compare communication quality and mental health outcome differences among the classes of LBC and between each of the classes and never-LBC.

**Results:**

Five latent classes of communication formed through different media or channels between migrant parents and their LBC were identified. Higher household economic status (OR = 2.81, *p* < 0.05) was associated with adequate communication. LBC in Class 1, defined by frequent technologically-mediated and face-to-face communication, had a significantly higher quality of communication with their migrant parents (F = 8.92, *p* < 0.001) and better mental health than those in other latent classes; these children did not have significantly worse mental health outcomes compared to never -LBC.

**Conclusions:**

Facilitating multichannel parent‒child communication is a practical way of reducing mental health inequities between LBC and their peers.

**Supplementary Information:**

The online version contains supplementary material available at 10.1186/s12889-024-17793-7.

## Introduction

Labor migration has become a central dynamic in the process of globalization and urbanization [1]. Even though migration often entails improved economic conditions for migrant families, it may also lead to increased family instability [2]. Because of institutional restrictions or unaffordable living costs in host countries or cities, many children are left behind by their migrant parents at their original residences; these children are also called left-behind children (LBC). A handful of studies have noted that compared to their peers who have never experienced parental migration (never-LBC), LBC might be exposed to more mental health risks, such as lower psychological resilience [3], more behavioral problems [4], suicidal ideation and suicide attempts [5]. Inadequate physical and psychological care from parents and inadequate parent–child communication brought by parental absence have been regarded as critical reasons accountable for the high mental health risks among LBC [6, 7]. Moreover, compared to children with one or no migrating parent, those who are left behind by both parents might be the most vulnerable with regard to mental health [7]. Also, the Chinese central government clearly defines its responsibility in protecting this group of children in national policies, such as the *Opinions of the State Council on Strengthening the Work of Caring for the Left-Behind Children in Rural Areas*. Therefore, with a special focus on Chinese children who are left behind by both parents, the current study aimed to explore the associations between the patterns of parent–child communication and their mental health outcomes.

Extensive studies have demonstrated the importance of parent‒child communication for children’s mental health [8]. Recently, increasing attention has been paid to communication between LBC and their migrant parents [9, 10]. Most extant studies have mainly focused on the quality or frequency of communication and have shown that a higher quality or frequency of communication is associated with better mental health outcomes among children, such as greater psychological resilience, fewer behavioral problems, and lower probabilities of nonsuicidal self-injury (NSSI) and suicidal ideation [4, 6, 11]. Nevertheless, few studies have evaluated the different effects of parent‒child communication established through different channels or media in migrant families with LBC.

In fact, LBC and migrant parents maintain their communication via multiple channels or media simultaneously, including technologically-mediated communication, such as phone/video calls, text messaging, and social media interaction, and face-to-face communication, such as parental home visits and children visiting their parents’ workplaces. Due to the progress in information technologies, the academic focus has recently shifted from inadequate face-to-face communication to the compensatory role of technologically-mediated communication between LBC and migrant parents [12]. The extant research on technologically-mediated communication has mainly utilized qualitative designs to investigate migrant parents’ experiences of long-distance parenting through phone/video calls and their perceived effectiveness in fulfilling parenting duties and maintaining positive parent‒child relationships across distances [9, 10, 13, 14]. However, the differences in the effects of technologically-mediated communication and face-to-face communication on the mental health of LBC remain unknown. More importantly, previous studies have commonly focused on individual communication channel variables separately, which ignored the clustering of cooccurring forms of communication.

Theoretically, rather than being independent of each other, different communication channels or media may have complex and interactive relationships. According to the cues-filtered out theory, face-to-face communication is regarded as the “gold standard” of communication, especially in situations that are ambiguous, emotional, and important [15]. The media niche theory posits that communication media have unique features and occupy a “niche” in overall communication [16]. The emergence and development of technologically-mediated communication may diminish the need to meet face-to-face, which is called the displacement hypothesis [17]. Although theoretically plausible, there is a lack of empirical support for this hypothesis [18]. In contrast, the reinforcement hypothesis proposes that technologically-mediated communication is not superior to face-to-face communication, but it can complement or strengthen face-to-face communication [19], which has been supported by empirical studies on different close relationships, such as friendships and dating relationships [20, 21]. Furthermore, in support of the reinforcement hypothesis, the media complexity theory suggests that the more means are used in communication, the greater the communication and the closer the relationships will be [22]. However, relevant exploration of these theories is still lacking for parent‒child relationships, let alone communication between LBC and their migrant parents.

China represents a unique opportunity to explore the heterogeneity within the group of LBC with regard to parent‒child communication and mental health due to its considerable population of migrant families. Rapid urbanization and industrialization in China have been accompanied by a growing population of rural-to-urban migrants, which soared from 6.7 million in 1982 to 376 million in 2020 [23, 24]. Under the household registration (*hukou*) system in China, individuals are assigned to a hukou status according to their residential location, categorized as either rural or urban [25]. Public services and welfare are more accessible for individuals with urban *hukou* compared to those with rural *hukou* [26]. Consequently, the majority of migrant workers’ children, who typically hold rural *hukou*, are excluded from state-sponsored social welfare programs such as access to public schools in cities. Thus, thousands of Chinese children are left behind by one or both parents. According to the estimation based on the most current census data, there were 66.93 million LBC in rural China in 2020, accounting for 22.5% of the total population of Chinese children (0–17 years) [27, 28]. Among all LBC, 45.6% of them lived with neither parent [27].

With a special focus on Chinese children who are left behind by both parents, the current study aimed to explore the patterns of communication formed through different media or channels between LBC and their migrant parents using latent class analysis (LCA) and associated heterogeneities in the quality of communication and children’s mental health outcomes. As a person-centered modeling approach, LCA can be applied to identify unobserved, homogeneous, mutually exclusive groups or classes of individuals with common exposure patterns [29], allowing us to fully contextualize the ways LBC and migrant parents communicate with each other. LCA also permits the incorporation of more factors compared to traditional regression models with interaction terms, while offering good interpretability [30].

Specifically, the current study aimed to answer the following five research questions: (1) What are the differences in the quality of parent‒child communication and mental health outcomes between LBC and never-LBC, including psychological resilience, behavioral problems, prosocial behavior, NSSI, and suicidal ideation? (2) What are the latent patterns of communication formed through different media or channels between migrant parents and LBC? (3) What sociodemographic characteristics are associated with the assignment of LBC to a certain latent class? (4) What are the differences in the quality of parent‒child communication and mental health outcomes of LBC across latent classes? (5) What are the differences in the quality of parent‒child communication and mental health outcomes between the latent classes of LBC and never-LBC? Drawing from findings of previous studies [3–6], we hypothesized that LBC would have lower communication quality with their migrant parents, lower psychological resilience, more behavioral problems, less prosocial behavior, higher probabilities of having NSSI and suicidal ideation compared to never-LBC. Hypotheses were not formed for the rest of the research questions because the current study on the latent patterns of parent–child communication is completely exploratory.

## Methods

### Participants

We recruited participants from two counties (Nanling and Wuwei) in Anhui Province in central China. Anhui is one of the largest migrant-sending provinces, with 22.2% of the total population migrating domestically for employment [31]. In 2018, In Anhui, there were approximately 736,000 LBC with both parents migrating, accounting for 13.4% of total child population in Anhui and 10.6% of the total LBC in China [32]. Approximately 55% of the total population of Anhui lives in urban areas [33]. To reflect this urban‒rural split, random sampling was performed among urban and rural schools separately. Ten urban schools were randomly selected from the school list provided by the local Education Commission. Eight rural schools were chosen based on a multistage sampling scheme. Two townships from each county were randomly selected, and two schools were selected from each township. At each school, all children from the 5th to 8th grades (normally aged 11 to 14 years old, given that children can only start school when they reach the age of six) were sampled. Children from the 1st to 4th grades were excluded to ensure the literacy level for completing the questionnaires. The junior high schools did not consent to conduct the survey with 9th grade children because they were preparing for the high-school entry examination.

A total of 5,352 children were sampled, among which 5,291 (98.9%) children consented to take the survey. After removing the questionnaires of children who were reported by their teachers as having intellectual development abnormalities, severe mental illness, or physical disabilities, completed questionnaires were collected from 5,189 (98.1%) children. Considering that other forms of parental absence could also impact children’s mental health, children who reported their parents as being divorced or having passed away were removed from the current study (*n* = 357). Participants were asked to answer two separate questions regarding the migration status of their father and mother: “Has your father or mother taken a job away from your hometown and been absent for over six months?”. The options were “currently absent,” “previously absent, not currently” and “never.” Only those who answered “currently absent” were further asked to answer questions about how they communicated with their migrant parents. According to most previously LBC’s feedback in the pilot survey we conducted, they found it difficult to recall their communication with their parents, because it had been a long time ago. Children who answered “previously absent, not currently” (*n* = 1,330) were excluded because they were not asked about their communication with parents to avoid recall difficulty. Currently LBC with only one parent migrating (*n* = 1,319) were excluded to prevent the violation of the missing at random assumption of the LCA (Little’s test statistic = 989.00, *p*-value < 0.001). In the end, a total of 2,183 children were included in this study, including 1,025 LBC who were currently left behind by both parents and 1,158 children never-LBC. Among the total children we initially sampled (*N* = 5,352), 19.2% were LBC currently left behind by both parents (*n* = 1,025). The two counties can be considered typical migrant-sending counties in Anhui, as this ratio is higher than the recent official data for the entire province (13.4%).

### Measures

#### Communication between LBC and their migrant parents

LBC were asked to answer five questions about how they communicated with migrant father and the same five questions about how they communicated with migrant mother. Of the five questions, two covered frequencies of technologically-mediated communications via audio phone calls and video calls in the past month. LBC were asked to select one of five options: “never”, “1–4 times”, “5–9 times”, “10–14 times”, and “more than 14 times”. The other three questions were about face-to-face communication in the last year. LBC were asked to recall whether their parents came back home during the Spring Festival (the Chinese Lunar New Year); how many times their parents had come back home at the original residences; and whether they visited their parents at their workplaces during summer/winter vacations. To conduct the LCA, all ten items were dichotomized [34]. Besides the natural split according to yes/no responses, the dichotomizations were based on a 50% percentile split for questions regarding the frequencies of technologically-mediated communications and on a median split for the number of times migrant parents visited home.

#### Quality of parent‒child communication

The quality of parent‒child communication was assessed using the Parent-Adolescent Communication Scale (PACS) [35]. The PACS includes two subscales: the open family communication subscale (10 items) and the problems in family communication subscale (10 items). Each item is scored on a 5-point Likert scale ranging from 1 (strongly disagree) to 5 (strongly agree). The total PACS score ranges from 20 to 100, with higher scores indicating higher quality of communication. The PACS has been proven to have good internal consistency (Cronbach’s α: 0.84 for mothers and 0.87 for fathers) in Chinese children [36]. Cronbach’s α was 0.84 for mothers and 0.87 for fathers for the PACS in the current study.

#### Psychological resilience

This study measured children’s resilience using the 25-item Connor-Davidson Resilience Scale (CD-RISC) [37]. Each item in the CD-RISC is assessed using a 5-point Likert scale. Within the range of 0 ~ 100, a higher total score indicates greater psychological resilience. Cronbach’s α was 0.91 for the Chinese version of the CD-RISC [38]. Cronbach’s α was 0.92 for the CD-RISC in this study.

#### Strengths and difficulties

To measure children’s psychiatric properties, the widely adopted Strengths and Difficulties Questionnaire (SDQ) [39] was used. The SDQ is a 25-item scale composed of five subscales: emotional problems, conduct problems, hyperactivity, peer problems, and prosocial behavior. The sum of the first four subscales yields a score for difficulties. Responses are in a Likert-type format with three options (0 = “Not true”, 1 = “Somewhat true”, 2 = “Certainly true”). The total difficulties score ranges from 0 to 40, and the prosocial behavior score ranges from 0 to 10, with higher scores indicating higher difficulties and more prosocial behavior. The Chinese version of the SDQ was proven to be valid and reliable [40]. In the current study, Cronbach’s α was 0.74 for difficulties and 0.67 for prosocial behavior.

#### Nonsuicidal self-injury

Nonsuicidal self-injury (NSSI) was measured with a self-designed question: “Have you hurt yourself deliberately during the past year (including self-cutting, scratching, jumping from heights, overdosing on drugs, swallowing indigestible things, etc.)?” Three options were available: “no,” “once,” and “more than once.” Participants who answered “once” or “more than once” were defined as having NSSI (coded as 1), and those who answered “no” were defined as not having NSSI (coded as 0) [41].

#### Suicidal ideation

Suicidal ideation was measured using the suicide item of the Beck Depression Inventory (BDI) [42]. The BDI is a classic 21-item self-report scale for measuring depressive symptoms. Answer options for the suicide item were (1) “I don’t have any thoughts of killing myself”; (2) “I have thoughts of killing myself, but I would not carry them out”; (3) “I would like to kill myself”; and (4) “I would kill myself if I had the chance.” Participants who selected options 2 to 4 were defined as having suicidal ideation (coded as 1), and those who selected other options were defined as not having suicidal ideation (coded as 0). The suicidal ideation item in the Chinese version of the BDI was characterized by good validity and was used in previous studies on Chinese LBC [43, 44].

#### Sociodemographic variables

The sociodemographic variables included children’s age, gender, residency, parental education level, only child status, household income status, and number of friends, which have been commonly used as covariates in previous studies on Chinese LBC [4, 6, 7, 44]. Household income status was measured with an objective item (total count of electronic devices and vehicles, ranging from 0 to 9) [45] and a subjective item (perceived economic status relative to other households in the neighborhood).

### Statistical analyses

A total of 1.3%-13.8% of the responses contained missing values for sociodemographic variables and outcome variables. A summary of the percentages of missing values can be found in Appendix 1 in the Supplementary document. Based on a missing at random assumption (Little’s test statistic = 146.0, *p* = 0.147), multiple imputation was used to fill in missing data for these variables.

First, descriptive analyses included sample counting and percentages of categorical variables, median and Interquartile Range (IQR) of count variables, and mean values and standard deviations of continuous variables. To compare the differences in the socio-demographics and mental health outcomes between LBC and never-LBC, chi-square tests were performed on categorical variables, Mann–Whitney U Test was conducted on count variables, and t-tests were conducted on continuous variables.

Second, the LCA was performed on the ten binary communication items (five items for each parent) to identify different communication patterns between LBC and their migrant parents. To address missing values in the ten communication items used in the LCA, full information maximum likelihood estimation was used instead. This method allows participants to be included as long as they responded to at least one of the ten communication items. To identify the best number of latent classes, model fit criteria, including the Bayesian information criterion (BIC), the adjusted BIC, Akaike’s information criterion (AIC), and entropy, were compared across models with two to six classes. A model with a minimum BIC, a-BIC, and AIC was desired. Entropy values over 0.8 indicated good separation of classes, with higher entropy indicating better fit. Since different criteria might point to different optimal numbers of classes, class proportions and subjective interpretability were also considered when making the final choice.

Third, a multinomial logistic regression analysis was conducted to assess the associations between sociodemographic variables and class membership of LBC.

Fourth, to examine the internal difference in communication quality and mental health outcomes within LBC, analysis of covariance (ANCOVA) was performed on continuous outcomes, controlling for sociodemographic variables. Following significant ANCOVA F statistics, Tukey’s tests for post hoc analyses were conducted to make pairwise comparisons. Tests for normality of the continuous outcomes were conducted with Q-Q (quantile–quantile) plots. For categorical outcome variables, chi-square tests were performed. Following significant chi-square tests, pairwise proportion comparisons were conducted. While covariates were not incorporated in chi-square tests, we conducted a comparative analysis of results with covariates using logistic regression and without covariates using chi-square tests. The outcomes were found to be similar. For the sake of consistency in reporting, we chose to present the results of the chi-square tests.

Fifth, the communication quality and mental health outcomes of the latent classes within the LBC were further compared with those of the never-LBC group using ANCOVA and chi-square tests. Dunnett’s tests were used for post-hoc pairwise comparisons.

In addition, to examine the robustness of our findings, a sensitivity analysis was conducted. As the majority of parents returned home during the Spring Festival, we removed “home visit during the Spring Festival” of both parents from the indicators of latent classes and then repeated the LCA on eight binary communication variables.

R Studio version 1.3.1093 was used to conduct the preliminary data cleaning, descriptive analyses, and regression analyses. M-plus 8.3 was used to complete the LCA. A *p* value less than 0.05 was assumed to be statistically significant.

### Ethical considerations

The survey was conducted from April 2018 to March 2019. Prior to the survey, we contacted the school principals and obtained their consent. A letter of informed consent was distributed to each student to ask for their consents to participate in the survey and was taken home to obtain parental consent or other legal guardian. On the day of the survey, children who agreed to participate in the survey and whose parents gave their consent anonymously completed questionnaires in the classrooms. Each classroom survey was administered by two trained surveyors. Teachers were required to be absent from the classrooms during the whole process to guarantee children’s true and distraction-free responses. Ethical approval of this study was obtained from the Ethics Committee of Zhejiang University (project number: ZGL201804-2).

## Results

### Sample characteristics

As shown in Table 1, the average age of the whole sample (*N* = 2,183) was 12.95 years (SD = 1.29). The average age of the LBC group (*n* = 1,025; female% = 43.7) was 13.13 years (SD = 1.26) compared to that of 12.80 years (SD = 1.29) for the never-LBC group (*n* = 1,158; female% = 44.8), and the difference was statistically significant (t = -6.00, *p* < 0.001). Significant differences were observed in the residency type (χ^2^ = 445.43, *p* < 0.001) and parental education level (fathers: χ^2^ = 87.26, *p* < 0.001; mothers: χ^2^ = 48.34, *p* < 0.001) of the LBC and never-LBC groups. Specifically, more than half of the LBC (68.2%) in our sample were from rural areas, and most of the never-LBC (76.9%) lived in urban areas. The education level of the parents of LBC was mostly middle school and lower (84.1% fathers, 87.4% mothers), and only a few parents had college degrees or higher (3.3% for both parents). Comparatively, more parents of never-LBC had college degrees or higher (11.1% fathers, 9.5% mothers). Regarding household income status, although LBC and never-LBC reported similar perceptions (χ^2^ = 1.38, *p* = 0.50), the households of never-LBC had more electronic devices and vehicles (mean = 7.24, SD = 1.24) than those of LBC (mean = 6.85, SD = 1.40; t = 6.84, *p* < 0.01). LBC and never-LBC showed no significant difference in only child status or the number of friends.

**Table 1:** Sample characteristics

Variable	Whole sample (*N* = 2183)	LBC (*n* = 1025)	Never-LBC (*n* = 1158)	Differences between LBC and never-LBC
	Mean (SD) / N (%) / Median (IQR)	Mean (SD) / N (%) / Median (IQR)	Mean (SD) / N (%) / Median (IQR)	T / χ2 / W
**Socio-demographic characteristics**				
Age	12.95 (1.29)	13.13 (1.26)	12.80 (1.29)	t = -6.00***
Gender				χ2 = 0.23
Male	1216 (55.7%)	577 (56.3%)	639 (55.2%)	
Female	967 (44.3%)	448 (43.7%)	519 (44.8%)	
Residency				χ2 = 445.43***
Rural	967 (44.3%)	699 (68.2%)	268 (23.1%)	
Urban	1216 (55.7%)	326 (31.8%)	890 (76.9%)	
Father’s education				χ2 = 87.26***
College and higher	163 (7.5%)	34 (3.3%)	129 (11.1%)	
High school	374 (17.1%)	129 (12.6%)	245 (21.2%)	
Middle school and lower	1646 (75.4%)	862 (84.1%)	784 (67.7%)	
Mother’s education				χ2 = 48.34***
College and higher	144 (6.6%)	34 (3.3%)	110 (9.5%)	
High school	254 (11.6%)	95 (9.3%)	159 (13.7%)	
Middle school and lower	1785 (81.8%)	896 (87.4%)	889 (76.8%)	
Only child	729 (33.4%)	335 (32.7%)	394 (34.0%)	χ2 = 0.38
Perceived economic status				χ2 = 1.38
Poor	134 (6.1%)	64 (6.2%)	70 (6.0%)	
Fair	1422 (65.1%)	679 (66.2%)	743 (64.2%)	
Wealthy	627 (28.7%)	282 (27.5%)	345 (29.8%)	
Electronic devices and transportation	7.06 (1.33)	6.85 (1.40)	7.24 (1.24)	t = 6.84***
Number of friends	4 (4)	4(4)	4(4)	W = 595,430
**Quality of communication**				
Father-child communication	58.04 (10.30)	57.47 (10.19)	58.55 (10.37)	t = 2.44*
Mother–child communication	58.97 (11.02)	57.73 (10.84)	60.07 (11.08)	t = 4.97***
**Mental health outcomes**				
Psychological resilience	59.28 (16.45)	57.56 (16.45)	60.80 (16.32)	t = 4.60***
Difficulties	12.04 (5.53)	12.65 (5.48)	11.50 (5.51)	t = -4.87***
Prosocial behavior	7.05 (2.03)	6.95 (2.01)	7.14 (2.04)	t = 2.23*
Nonsuicidal self-injury	290 (13.3%)	158 (15.4%)	132 (11.4%)	χ2 = 7.22**
Suicidal ideation	524 (24.0%)	265 (25.9%)	259 (22.4%)	χ2 = 3.10

*LBC* left-behind children

^*^p < 0.05; **p < 0.01; ***p < 0.001

### Differences in the quality of communication and mental health outcomes between LBC and never-LBC

As shown in Table 1, LBC had lower quality father-child (t = 2.44, *p* < 0.05) and mother–child (t = 4.97, *p* < 0.001) communication than never-LBC. Regarding mental health outcomes, compared to never-LBC, LBC had lower psychological resilience (t = 4.60, *p* < 0.001) and less prosocial behavior (t = -4.87, *p* < 0.001), more difficulties (t = 2.23, *p* < 0.05) and a larger proportion of having NSSI (χ^2^ = 7.22, *p* < 0.01). No significant difference was found in the proportion of having suicidal ideation between LBC and never-LBC (χ^2^ = 3.10, = 0.07).

### Latent patterns of communication between LBC and their migrating parents

In terms of the characteristics of parent‒child communication for the entire group of LBC, most LBC reported that they had talked with their migrant parents via phone more than 4 times in the past month (father-child communication: 65.7%; mother–child communication: 70.0%). Some LBC reported that they had talked with their fathers (34.5%) or mothers (41.5%) via video more than 4 times in the past month. On average, migrant parents returned home to their original residences 3–4 times during the last year (fathers: mean = 3.76, SD = 5.31; mothers: mean = 3.73, SD = 5.65). Approximately 80% of the LBC had visited their parents’ workplaces during summer or winter vacations in the last year. Over 90% of migrant parents returned home during the Spring Festival. Table 2 presents the summary statistics of the ten communication items.

**Table 2:** Summary statistics of ten communication items of LBC

**Variable**	**Father-child communication** Mean (SD)/N (%)	**Mother–child communication** Mean (SD)/N (%)
**Technologically-mediated communication in the past month**		
Phone call frequency		
Never	71 (7.0%)	45 (4.4%)
1–4 times	278 (27.3%)	261 (25.6%)
5–9 times	217 (21.3%)	233 (22.8%)
10–14 times	161 (15.8%)	178 (17.5%)
15 times and more	291 (28.6%)	303 (29.7%)
Video call frequency		
Never	392 (38.5%)	336 (33.1%)
1–4 times	275 (27.0%)	257 (25.3%)
5–9 times	129 (12.7%)	161 (15.8%)
10–14 times	86 (8.5%)	99 (9.7%)
15 times and more	135 (13.3%)	163 (16.0%)
**Face-to-face communication in the past year**		
Frequency of parental home visit	3.76 (5.31)	3.73 (5.65)
Home visits during the Spring Festival		
Yes	952 (93.3%)	947 (93.0%)
No	68 (6.7%)	71 (7.0%)
Children visiting at their parents’ workplaces during summer/winter vacations		
Yes	781 (76.5%)	815 (80.3%)
No	240 (23.5%)	200 (19.7%)

*LBC* left-behind children

Table 3 displays the fit indices for each LCA model, which helped to identify the best number of latent patterns or classes of communication between LBC and their migrating parents. The AIC, BIC, and a-BIC declined as the number of classes increased. The entropy values of all models were higher than 0.85, indicating higher accuracy of the classification of individuals in these models. The five-class model had the highest entropy (0.90) among all models. Although the AIC, BIC, and a-BIC indicated that the 6-class model should be chosen, the entropy and class proportion did not support this selection. One class in the six-class model accounted for only 5% of the total sample (*n* = 51), which might not provide reliable estimates of class-specific parameters in later analyses. Regarding models other than the six-class model, the five-class model had the lowest AIC, BIC, and a-BIC. Therefore, the five-class model was ultimately chosen. Five different latent patterns of communication formed through different media or channels between migrant parents and LBC were identified.

**Table 3:** LCA model fit index

Model	AIC	BIC	aBIC	Entropy	Class proportions
2-class	10533.01	10636.59	10569.89	0.856	0.35/0.65
3-class	10160.4	10318.24	10216.6	0.863	0.50/0.19/0.31
4-class	9864.685	10076.78	9940.208	0.871	0.28/0.17/0.19/0.36
5-class	9719.754	9986.106	9814.596	0.902	0.25/0.07/0.20/0.33/0.15
6-class	9606.584	9927.193	9720.747	0.882	0.17/0.21/0.11/0.05/0.31/0.15

*LCA* latent class analysis, *AIC* Akaike’s information criterion, *BIC* Bayesian information criterion

**Fig. 1:**
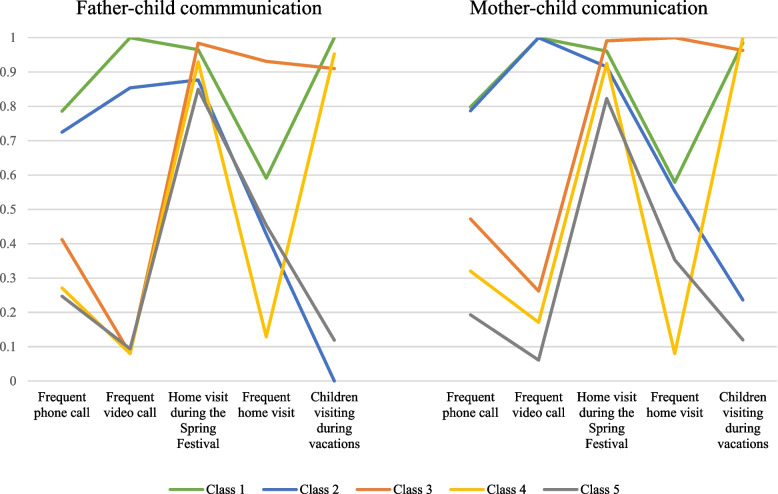
Class probability based on parent-child communication. Note: Class 1: Multichannel Communication; Class 2: Technologically-mediated Dominant Communication; Class 3: Frequent Parental Home Visits; Class 4: Children Visiting at Their Parents’ Workplaces; Class 5: Inadequate Communication

Figure 1 illustrates the item probabilities for each pattern or class. LBC demonstrated similar patterns when communicating with their fathers and mothers. Specifically, Class 1 (*n* = 255, 24.9%) was termed *Multichannel Communication.* The prominent feature of this class is the highest probability of having frequent technologically-mediated communication via phone or video calls and a fairly high probability of having frequent face-to-face communication. Class 2 (*n* = 68, 6.6%) was labeled *Technologically-mediated Dominant Communication* because members of this class had a high probability of frequent technologically-mediated communication but a low probability of engaging in face-to-face communication, such as parental home visit and children visiting parents’ workplaces. Compared to Class 1 and Class 2, the remaining three classes of children had significantly lower probabilities of having frequent technologically-mediated communication. Class 3 (*n* = 204, 19.9%) was termed *Frequent Parental Home Visits*, as members in this class demonstrated the highest probability of having frequent parental home visits. The prominent feature of the LBC in Class 4 (*n* = 345, 33.7%) was the high probability of visiting their parents during vacations, but their parents were the least likely to return home. Thus, this class was termed *Children Visiting at Their Parents’ Workplaces*. Class 5 (*n* = 153, 14.9%) was labeled *Inadequate Communication* because LBC in this class had the lowest probability of technologically-mediated communication and a low probability of meeting with their parents in person. Finally, both mothers and fathers had very high probabilities of returning home during the Spring Festival regardless of class. Between-class differences for each indicator are shown in Appendix 2 of the Supplementary document.

### Associations between sociodemographic factors and class membership

Table 4 demonstrates the associations between LBC’s sociodemographic characteristics and their odds of being assigned to Classes 1–4 relative to Class 5. Younger LBC had higher odds of being assigned to Class 1 (β = -0.21, 95% CI [-0.39, -0.04], OR = 0.81, *p* < 0.05) and Class 3 (β = -0.19, 95% CI [-0.37, -0.01], OR = 0.83, *p* < 0.05) relative to Class 5. Compared to urban LBC, rural LBC had a lower probability of being in Class 2 compared to Class 5 (β = -1.35, 95% CI [-1.97, -0.73], OR = 0.26, *p* < 0.001). Children whose mothers had a college education or higher were associated with lower odds of being assigned to Class 1 (β = -1.32, 95% CI [-2.59, -0.06], OR = 0.27, *p* < 0.05) and Class 3 (β = -1.68, 95% CI [-3.18, -0.18], OR = 0.19, *p* < 0.05) compared to Class 5. Compared to LBC who perceived their household economic status as poor, those who perceived their households as wealthy were 2.81 times more likely to be in Class 1 (β = 1.03, 95% CI [0.10, 1.97], OR = 2.81, *p* < 0.05) and 4.23 times more likely to be in Class 3 (β = 1.44, 95% CI [0.29, 2.59], OR = 4.23, *p* < 0.05) relative to being in Class 5. A higher number of owned electronic devices and vehicles was associated with a higher probability of being assigned to Class 1 (β = 0.51, 95% CI [0.34, 0.67], OR = 1.65, *p* < 0.001) and Class 3 (β = 0.49, 95% CI [0.33, 0.66], OR = 1.64, *p* < 0.001). Finally, LBC with more friends were more likely to be assigned to Class 1 (β = 0.05, 95% CI [0.00, 0.10], OR = 1.05, *p* < 0.05) and Class 4 (β = 0.05, 95% CI [0.00, 0.10], OR = 1.05, *p* < 0.05) than to Class 5.

**Table 4:** The associations between sociodemographic factors and class membership

Reference group: Class 5 (*n* = 153)	Class 1 (*n* = 255)			Class 2 (*n* = 68)			Class 3 (*n* = 204)			Class 4 (*n* = 345)		
	β	OR	95% CI	β	OR	95% CI	β	OR	95% CI	β	OR	95% CI
Age	-0.21*	0.81	[-0.39, -0.04]	-0.21	0.81	[-0.44, 0.03]	-0.19*	0.83	[-0.37, -0.01]	0.02	1.02	[-0.14, 0.18]
Gender: Female (ref: male)	0.11	1.11	[-0.33, 0.54]	0.00	1.00	[-0.60, 0.61]	0.07	1.07	[-0.38, 0.51]	-0.17	0.84	[-0.57, 0.23]
Residency: rural (ref: urban)	-0.44	0.65	[-0.90, 0.03]	-1.35***	0.26	[-1.97, -0.73]	0.18	1.20	[-0.32, 0.68]	-0.06	0.94	[-0.49, 0.38]
Father's education (ref: Middle school and below)												
College and above	0.66	1.94	[-0.76, 2.09]	0.61	1.84	[-1.12, 2.35]	0.28	1.32	[-1.34, 1.90]	-0.32	0.72	[-1.82, 1.18]
High school or secondary technical school	-0.12	0.88	[-0.79, 0.54]	0.32	1.38	[-0.53, 1.17]	-0.29	0.75	[-1.00, 0.42]	-0.38	0.68	[-1.03, 0.27]
Mother's education (ref: Middle school and below)												
College and above	-1.32*	0.27	[-2.59, -0.06]	-0.53	0.59	[-2.09, 1.03]	-1.68*	0.19	[-3.18, -0.18]	-0.85	0.43	[-2.05, 0.36]
High school or secondary technical school	-0.13	0.88	[-0.89, 0.63]	-0.01	0.99	[-1.00, 0.99]	-0.53	0.59	[-1.38, 0.32]	-0.26	0.77	[-1.01, 0.49]
Only child (ref: no)	0.12	1.12	[-0.34, 0.57]	-0.15	0.86	[-0.82, 0.51]	0.21	1.24	[-0.25, 0.68]	-0.11	0.90	[-0.53, 0.32]
Perceived economic status (ref: poor)												
Fair	0.35	1.42	[-0.49, 1.19]	-0.02	0.98	[-1.13, 1.10]	1.17*	3.21	[0.11, 2.23]	0.24	1.27	[-0.44, 0.92]
Wealthy	1.03*	2.81	[0.10, 1.97]	0.60	1.82	[-0.63, 1.82]	1.44*	4.23	[0.29, 2.59]	0.52	1.68	[-0.28, 1.32]
Availability of electronic devices and vehicles	0.50***	1.65	[0.34, 0.67]	0.21	1.24	[-0.02, 0.44]	0.49***	1.64	[0.33, 0.66]	0.09	1.09	[-0.05, 0.23]
Number of friends	0.05*	1.05	[0.00, 0.10]	0.04	1.04	[-0.01, 0.09]	0.04	1.04	[-0.01, 0.09]	0.05*	1.05	[0.00, 0.10]

*OR* odds ratio

^*^p < 0.05; **p < 0.01; ***p < 0.001

### Differences among the latent classes of LBC

**Fig. 2:**
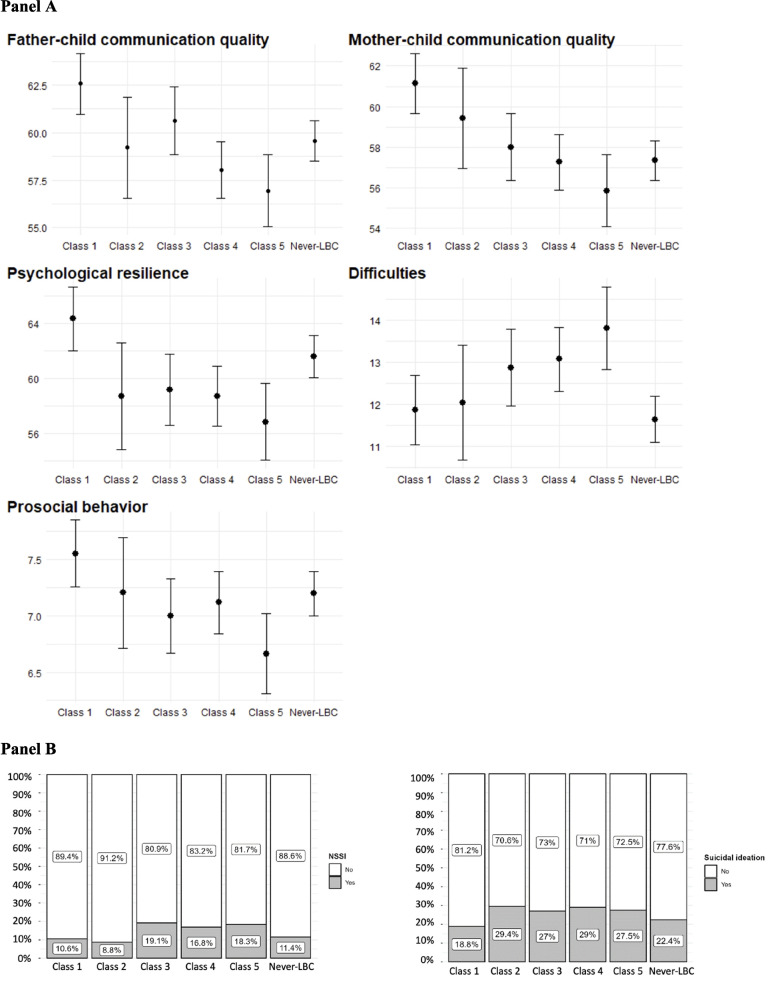
Communication quality and mental health outcomes of classes of LBC and never-LBC. *Note: *LBC=left-behind children; NSSI = Nonsuicidal self-injury

Continuous variables met the assumption of normality, with results shown in Appendix 3 of the Supplementary document. As shown in Table 5 and Fig. 2, there were significant differences among the five latent classes in terms of the quality of all communication and mental health outcomes except suicidal ideation. The results of post-hoc pairwise comparisons showed that both Class 1 (adjusted mean = 62.58, SE = 0.81) had significantly higher (F = 9.71, *p* < 0.001) father-child communication quality than Class 4 (adjusted mean = 58.05, SE = 0.75) and Class 5 (adjusted mean = 56.95, SE = 0.97). Class 3 (adjusted mean = 60.62, SE = 0.91) also had better father-child communication than Class 5. LBC in Class 1 (adjusted mean = 61.14, SE = 0.76) had better (F = 8.92, *p* < 0.001) communication with their mothers than LBC in Class 3 (adjusted mean = 58.00, SE = 0.84), Class 4 (adjusted mean = 57.27, SE = 0.70), and Class 5 (adjusted mean = 55.85, SE = 0.91). Regarding mental health outcomes, children in Class 1 had significantly greater psychological resilience (adjusted mean = 64.32, SE = 1.19) than those in Class 3, Class 4, and Class 5 (F = 8.05, *p* < 0.001). Moreover, scores on the SDQ showed that children in Class 1 had significantly fewer difficulties (adjusted mean = 11.86, SE = 0.42) than those in Class 5 (adjusted mean = 13.81, SE = 0.50; F = 3.65, *p* < 0.01) and more prosocial behavior (adjusted mean = 7.55, SE = 0.15) than those in Class 3 and Class 5 (F = 5.27, *p* < 0.001). Finally, Class 1 (10.6%) had a significantly lower proportion of having NSSI than Class 4 (16.8%) and Class 5 (18.3%; χ^2^ = 10.46, *p* < 0.05). Standardized effect sizes for the differences among latent classes are shown in Appendix 4 of the Supplementary document.

**Table 5:** Differences among the latent classes of LBC and between latent classes and never-LBC

Variable	Class 1 (*n* = 255)	Class 2 (*n* = 68)	Class 3 (*n* = 204)	Class 4 (*n* = 345)	Class 5 (*n* = 153)	ANCOVA^a^ or χ2 tests among latent classes of LBC		Never-LBC (*n* = 1158)	ANCOVA^a^ or χ2 tests between latent classes and never-LBC	
	Adjusted^a^ mean (standard error) or Count (percentage)					Test statistics	Post hoc analysis^b^	Adjusted mean^a^ (standard error) or Count (percentage)	Test statistics	Post hoc analysis^c^
Father-child communication	62.58 (0.81)	59.21 (1.35)	60.62 (0.91)	58.05 (0.75)	56.95 (0.97)	9.71***	1 > 4; 1 > 5; 3 > 5	59.58 (0.54)	7.41***	1 > never-LBC
Mother–child communication	61.14 (0.76)	59.42 (1.26)	58.00 (0.84)	57.27 (0.70)	55.85 (0.91)	8.92***	1 > 3; 1 > 4; 1 > 5	57.34 (0.50)	7.86***	1 > never-LBC
Psychological resilience	64.32 (1.19)	58.67 (1.98)	59.15 (1.33)	58.69 (1.11)	56.81 (1.43)	8.05***	1 > 3; 1 > 4; 1 > 5	61.57 (0.79)	17.00***	never-LBC > 5
Difficulties	11.86 (0.42)	12.03 (0.70)	12.86 (0.47)	13.06 (0.39)	13.81 (0.50)	3.65**	1 < 5	11.63 (0.28)	8.45***	never-LBC < 4; never-LBC < 5
Prosocial behavior	7.55 (0.15)	7.20 (0.25)	7.00 (0.17)	7.12 (0.14)	6.66 (0.18)	5.27***	1 > 3; 1 > 5	7.20 (0.10)	8.97***	never-LBC > 5
Nonsuicidal self-injury	27 (10.6%)	6 (8.80%)	39 (19.10%)	58 (16.80%)	28 (18.3%)	10.46*	1 < 4; 1 < 5	132 (11.4%)	19.40**	never-LBC < 3 never-LBC < 4 never-LBC < 5
Suicidal ideation	48 (18.8%)	20 (29.4%)	55 (27.00%)	100 (29.0%)	42 (27.5%)	9.12	-	259 (22.4%)	13.17*	never-LBC < 5

*LBC* left-behind children

^*^p < 0.05; **p < 0.01; ***p < 0.001

^a^Adjusted for children’s age, gender, residency, parental education level, only child status, household income status, and number of friends

^b^Tukey’s tests were used for continuous variables and chi-square independence test was used for categorical variables

^c^Dunnett's test were used for continuous variables and chi-square independence test was used for categorical variables

### Differences between the latent classes of LBC and never-LBC

As shown in Table 5 and Fig. 2, significant differences in the quality of all communication and mental health outcomes were detected between each of the latent classes and never-LBC. The results of Dunnett's tests showed that LBC in Class 1 had higher quality father-child (adjusted mean difference [AMD] = 2.99, 95% CI [1.03, 4.96], *p* < 0.001) and mother–child (AMD = 3.80, 95% CI [1.97, 5.62], *p* < 0.001) communication than never-LBC. LBC in Class 5 (AMD = -4.76, 95% CI [-8.38, -1.14], *p* < 0.01) had significantly lower psychological resilience than never-LBC. Compared to never-LBC, LBC in Class 4 (AMD = 1.43, 95% CI [0.10, 2.36], *p* < 0.001), and Class 5 (AMD = 2.17, 95% CI [0.90, 3.44], *p* < 0.001) had more difficulties. In terms of prosocial behavior, only LBC in Class 5 had a lower score than never-LBC (AMD = -0.53, 95% CI [-0.99, -0.08], *p* < 0.05). LBC in Class 3 (19.1%), Class 4 (16.8%), and Class 5 (18.3%) had higher proportions of having NSSI than never-LBC (11.4%; *p* < 0.05). LBC in Class 5 (27.5%) had a higher proportion of having suicidal ideation than never-LBC (22.4%; *p* < 0.05). Standardized effect sizes for the differences between never-LBC and latent classes are shown in Appendix 4 of the Supplementary document.

### Results of the sensitivity analysis

Excluding “home visits during the Spring Festival” of both mothers and fathers as indicators in the sensitivity analysis, the new model fit indices supported the selection of a five-class model as the optimal number of parent‒child communication patterns. One difference in the results was observed. The test statistic in the result of the chi-square test for NSSI equaled 9.00, becoming insignificant after the exclusion of home visits during the Spring Festival. Though the result of the chi-square test was very close to the main analysis result (χ^2^ = 10.46, *p* < 0.05), caution is required when generalizing the finding concerning NSSI. The results of the sensitivity analysis are displayed in the Appendix 5 of the Supplementary document.

## Discussion

Parental migration has imposed great psychological challenges on millions of LBC around the world [5, 46]. With special attention to Chinese children whose parents both migrated, the current study is among the first to reveal the communication patterns between LBC and their migrant parents and explore the associated differences in the quality of communication and LBC’s mental health outcomes. By conducting LCA for quantitative data, this study identified five subgroups of LBC characterized by unique communication patterns with their migrant parents. These groups differed in both the common use of media or channels of communication and the person who initiated the communication. Compared to traditional methods, such as natural categorization, LCA offers a more person-centered and data-driven approach, enabling us to uncover complex, latent patterns in parent–child communication that might otherwise remain hidden in less sophisticated categorizations. The results of this study showed differences in the quality of parent‒child communication and mental health outcomes among the five subgroups of LBC and between LBC in each of the subgroups and never-LBC.

Consistent with previous studies [4, 5], the current study found that LBC as a whole were more disadvantaged regarding mental health than never-LBC, with LBC having lower resilience, more behavioral problems, fewer prosocial behaviors, and an even higher tendency toward NSSI. Parental migration might directly contribute to this gap in mental health, as parental absence leads to family instability and nonintact family structures [2]. More importantly, this gap in mental health might also reveal a deep-rooted structural health inequity among Chinese children. The descriptive statistics in our study showed significant differences in the sociodemographic characteristics between LBC and never-LBC. Specifically, LBC were more likely to live in rural areas, had parents with low education levels, and were from poor families.

In fact, we found that the establishment of a certain pattern of communication between LBC and their migrant parents might also be constrained or influenced by sociodemographic factors, especially family socioeconomic status (SES). The results of the current study showed that LBC who lived in rural areas or were from poorer families were more likely to have inadequate communication (Class 5) with their migrant parents rather than frequent technologically-mediated communication (Class 1 and Class 2) and frequent parental home visits (Class 3). The relatively high cost of long-distance calling and difficulties in accessing free internet in rural areas might impede low-income migrant workers from communicating with their LBC [14]. Due to the vast territory of China, cross-regional travel requires much time and money, which might also impede low-income migrant workers from frequently returning home to visit their children. Potential difficulties in family relationships caused by the lack of parent‒child communication might make these rural LBC living in low-income families more vulnerable. In addition, we found that only the maternal education level was significantly associated with communication patterns, whereas the paternal education level was not. Females with college degrees might migrate to places farther from their hometown, potentially making it difficult for them to balance between work duties and maintaining good mother–child communication. However, the inferential power of the result was quite limited (0.03 < *p* < 0.05), as migrant mothers with a college degree or above accounted for only a very small proportion (3.3%) of the sample.

In this study, we investigated the differences in the quality of parent‒child communication and mental health outcomes within LBC and between the subgroups of LBC and never-LBC. In terms of the quality of communication, LBC in Class 1 (*Multichannel Communication*) had significantly higher quality communication with their migrant parents compared to not only children in other latent classes but also never-LBC. Although Class 2 (*Technologically-mediated Dominant Communication*) did not outperform the other latent classes, it did not have significantly worse performance compared to Class 1. In terms of mental health outcomes, Class 1 outperformed the other four classes of LBC. The mental health outcomes of Class 1 and Class 2 were not significantly worse than those of never-LBC. In contrast, LBC in either Class 3 (*Frequent Parental Home Visits*) or Class 4 (*Children Visiting at Their Parents’ Workplaces*) were more likely to have mental health risks than never-LBC. It is important to note that both Class 1 and Class 2 were defined by frequent technologically-mediated communication, and both Class 3 and Class 4 had a significantly lower probability of frequent technologically-mediated communication. In other words, without adequate technologically-mediated communication, face-to-face communication initiated either by migrant parents (Class 3) or LBC (Class 4) did not provide substantial advantages in mental health outcomes. These findings suggested that technologically-mediated communication between migrant parents and their LBC via audio phone calls and video calls had great potential in sustaining the quality of parent‒child communication and maintaining the mental health of LBC. In particular, the use of video calls could not only help family members keep track of each other’s whereabouts but also enable migrant parents and their LBC to actually “see” each other and compensate for the loss in intimacy and familiarity caused by physical absence [12, 47].

Echoing previous theories in communication, our findings might not support the displacement hypothesis [17] due to the prominent comparative advantages of Class 1, defined by frequent technologically-mediated and face-to-face communication. Although the emergence of technologically-mediated communication was very important for the quality of communication, it might not diminish the need of LBC and their migrant parents to meet face-to-face. As the “gold standard” of communication [15], face-to-face interactions can transmit verbal and nonverbal signals at the same time, which involves direct emotional support and effective parental supervision [48, 49]. Furthermore, the results of this study supported the reinforcement hypothesis and the media complexity theory [19, 22] in the context of parent‒child communication. By maintaining frequent interactions via diverse channels, LBC could make use of the benefits of different channels to better share their lives with their migrant parents, and parents could also provide emotional support [9, 13]. The use of technologically-mediated communication might reinforce or complement face-to-face communication between LBC and their migrant parents, which is consistent with findings based on other close relationships [18, 50].

Unquestionably, LBC in Class 5 (*Inadequate Communication*) were the most disadvantaged in terms of the quality of parent‒child communication and mental health outcomes compared to LBC in the other latent classes and never-LBC. These findings further highlight the importance of parent‒child communication for LBC. In addition, it was also observed that all five classes of LBC had very high probabilities of parental home visits during the Spring Festival. As a centuries-old tradition, Chinese families are supposed to reunite during the largest and most elaborate festival in China [51]. A previous ethnographic study revealed that family ritual activities during the Spring Festival, such as reunion meals, gift giving, and relative visits, could help migrant workers compensate for relational loss, adapt to familial roles, and transmit family values to their LBC [52]. However, our findings suggest that this once-a-year reunion might fail to compensate for the damage caused by prolonged family separation if these migrant families do not maintain frequent technologically-mediated communication.

### Implications

Health equity is at the core of the United Nations ‘Sustainable Development Goals’ overarching principle of leaving no one behind [53]. Health inequity is mostly driven by uneven distributions of resources that determine the material and psychosocial living conditions of children, which are unfair and avoidable [54]. Our findings revealed significant mental health differences among Chinese children, arising from the complex interplay among family socio-economic conditions, parental migration status, and patterns of parent–child communication. The realization of health equity between LBC and their peers would ultimately require action regarding the social determinants of health, such as income, education, and living environment [55]. Nevertheless, the action to facilitate changes in society is time-consuming and not achievable in the short term. Fortunately, the current study might contribute to a more practical way of closing the gap in mental health from the perspective of parent‒child communication. This study highlights that frequent technologically-mediated and face-to-face communication between migrant parents and their LBC could serve as a modifiable factor for protecting LBC from adverse mental health outcomes, especially when family separation is somewhat inevitable. Future interventions targeting LBC should work toward a parent‒child communication scheme encompassing diverse channels. More interventions for maintaining parent‒child communication, such as free access to the internet, low-cost communication devices, and home-visit subsidies, might be especially needed for LBC, especially those from low-income families or living in rural areas.

### Limitations and future research

Several limitations should be noted in the present study. First, findings from this cross-sectional research should be interpretated with caution. Experimental designs, such as randomized control trials, are needed to make causal inferences in future research. Second, the latent patterns found in this study might subject to limited external validity as we utilized a sample of children from two counties in a single province in China to conduct the LCA, and the two counties were not selected randomly. Generalizing the findings to other populations should be done cautiously, since the classification of parent–child communication found in the current study largely depends on the specific features of the sample population. Third, although this study specifically focused on LBC who were left behind by both parents to address the salient vulnerability of this group of children, it excluded previously LBC and currently LBC with only one migrant parent, who have also been reported to have greater mental health risks than never-LBC [7, 44]. Future studies are needed to explore the different parent‒child communication patterns among different types of migrant families. Moreover, this study did not include children in the 1st to 4th grades to ensure the literacy level for completing the questionnaires. Future research might also consider the inclusion of younger LBC by using child-centered, participatory approaches such as drawing-based approaches.

Fourth, self-report questionnaires were used in this study, which might induce bias and affect the results. Regarding the measurement of parent–child communication, several significant mediums such as text messaging and social media interactions were not considered. Moreover, the inquiries concerning face-to-face communications pertained to the previous year, yet some LBC’s parents had been away for less than a year, potentially leading to biases. Future research should aim for a more comprehensive and precise assessment of parent–child communication. To control for the length of the questionnaire and decrease participants’ burden, we utilized a single item to measure NSSI. Future studies could consider using well-established scales such as the NSSI subscale from the Self-harm Behavior Questionnaire [56]. Fifth, only some basic demographic characteristics of migrant families were examined as correlates of communication patterns, and the inferential power of maternal education level and the number of friends were weak (0.03 < *p* < 0.05). It is suggested that future studies further explore other potential factors, such as parental age, caregiving of grandparents, the distance of migration, the psychological well-being and media literacy of migrant parents, and the relationships between migrant parents and at-home caregivers [10]. Sixth, this study involved multiple hypothesis testing while examining the associations between class membership and socio-demographic variables, increasing the risk of encountering Type I error. Results with a *p*-value greater than 0.01 should be generalized with caution. To investigate the differences within LBC classes and between LBC and never-LBC, we employed chi-square tests for NSSI and suicidal ideation, facilitating pairwise comparisons. However, it should be noted that covariates could not be incorporated into the chi-square tests, a factor to consider in interpreting the results for NSSI and suicidal ideation. Finally, the current study only primarily studied the relationship between communication patterns and communication quality or LBC’s mental health outcomes. To gain a deeper understanding of the underlying mechanisms, it is suggested that future explorations examine potential mediators and moderators in the associations between communication patterns and the mental health outcomes of LBC. These could include factors such as the caregiving role of grandparents, the distance of migration, the psychological well-being and media literacy of migrant parents, and the relationships between migrant parents and at-home caregivers.

## Conclusion

To conclude, as an entire group, LBC are more disadvantaged with regard to the quality of parent–child communication and mental health outcomes than never-LBC in general. Nonetheless, parental migration alone might seldom have significant impacts on LBC’s well-being. The vulnerabilities of the LBC group can be explained by whether frequent technologically-mediated and face-to-face communication is sustained between migrant parents and their LBC. LBC who had frequent communication with parents via multiple channels had prominently higher quality parent‒child communication and better mental health than those in other latent classes. Facilitating multichannel parent‒child communication might be a practical way of reducing health inequities between LBC and their peers.

### Supplementary Information

**Additional file 1: Appendix 1.** Percentage of missing values. **Appendix 2.** Latent class odds ratio results. **Appendix 3.** QQ-plots# for checking the normality of continuous outcome variables. **Appendix 4.** Standardized effect sizes for the overall group differences. **Appendix 5.** Sensitivity analysis.

## Data Availability

The datasets generated and analysed during the current study are not publicly available due reasons of sensitivity but are available from the corresponding author on reasonable request.

## Abbreviations

LBC: Left-behind children
NSSI: Nonsuicidal self-injury
LCA: Latent class analysis
PACS: Parent-Adolescent Communication Scale
CD-RISC: Connor-Davidson Resilience Scale
SDQ: Strengths and Difficulties Questionnaire
BDI: Beck Depression Inventory
BIC: Bayesian information criterion
AIC: Akaike's information criterion
ANCOVA: Analysis of covariance
