# Supplementary material for: Associations of latent patterns of parent‒child communication with communication quality and mental health outcomes among Chinese left-behind children
Source: BMC Public Health. 2024 Jan 31;24:332. doi: 10.1186/s12889-024-17793-7 (PMC10829291; doi:10.1186/s12889-024-17793-7)
Supplement: Supplementary file 1 — Additional file 1: Appendix 1. Percentage of missing values. Appendix 2. Latent class odds ratio results. Appendix 3. QQ-plots# for checking the normality of continuous outcome variables. Appendix 4. Standardized effect sizes for the overall group differences. Appendix 5. Sensitivity analysis. [file 12889_2024_17793_MOESM1_ESM.docx]

Supplementary Document

Appendix 1. Percentage of missing values

Appendix 2. Latent class odds ratio results

Appendix 3. QQ-plots# for checking the normality of continuous outcome variables

Appendix 4. Standardized effect sizes for the overall group differences

Appendix 5. Sensitivity analysis

Appendix 1. Percentage of missing values

| Variable | Percentage of missing |
| --- | --- |
| **Socio-demographic characteristics** |  |
| Age | 0.00% |
| Gender | 0.00% |
| Residency | 0.00% |
| Father’s education | 13.79% |
| Mother’s education | 13.42% |
| Only child | 0.05% |
| Perceived economic status | 1.42% |
| Electronic devices and transportation | 0.27% |
| Number of friends | 3.21% |
| **Quality of communication** |  |
| Father-child communication | 2.52% |
| Mother-child communication | 3.53% |
| **Mental health outcomes** |  |
| Psychological resilience | 3.94% |
| Difficulties | 1.69% |
| Prosocial behavior | 0.64% |
| Nonsuicidal self-injury | 0.05% |
| Suicidal ideation | 0.05% |
| **Communication variables** |  |
| Phone call frequency with father | 0.68% |
| Video call frequency with father | 0.78% |
| Frequency of paternal home visit | 1.46% |
| Fathers' home visits during the Spring Festival | 0.49% |
| Children visiting at their mothers’ workplaces during summer/winter vacations | 0.39% |
| Phone call frequency with mother | 0.49% |
| Video call frequency with mother | 0.88% |
| Frequency of maternal home visit | 2.15% |
| Mothers' home visits during the Spring Festival | 0.68% |
| Children visiting at their mothers’ workplaces during summer/winter vacations | 0.98% |

**Appendix 2. Latent class odds ratio results**

| **Comparison** | **Indicator** | | **Odds ratio** | **S.E.** | **P-Value** | **Sig** |
| --- | --- | --- | --- | --- | --- | --- |
| Class 5 compared to Class 4 | Father-child communication | Frequent phone call | 0.88 | 0.22 | 0.59 |  |
|  |  | Frequent video call | 1.19 | 0.53 | 0.72 |  |
|  |  | Home visit during the Spring Festival | 0.43 | 0.19 | 0.00 | *** |
|  |  | Frequent home visit | 5.63 | 1.51 | 0.00 | *** |
|  |  | Children visiting during vacations | 0.01 | 0.00 | 0.00 | *** |
|  | Mother-child communication | Frequent phone call | 0.51 | 0.15 | 0.00 | *** |
|  |  | Frequent video call | 0.31 | 0.19 | 0.00 | *** |
|  |  | Home visit during the Spring Festival | 0.38 | 0.15 | 0.00 | *** |
|  |  | Frequent home visit | 6.26 | 6.66 | 0.43 |  |
|  |  | Children visiting during vacations | 0.00 | 0.00 | 999.00 |  |
| Class 5 compared to Class 3 | Father-child communication | Frequent phone call | 0.47 | 0.15 | 0.00 | *** |
|  |  | Frequent video call | 1.20 | 0.67 | 0.77 |  |
|  |  | Home visit during the Spring Festival | 0.09 | 0.10 | 0.00 | *** |
|  |  | Frequent home visit | 0.06 | 0.11 | 0.00 | *** |
|  |  | Children visiting during vacations | 0.01 | 0.01 | 0.00 | *** |
|  | Mother-child communication | Frequent phone call | 0.27 | 0.08 | 0.00 | *** |
|  |  | Frequent video call | 0.18 | 0.11 | 0.00 | *** |
|  |  | Home visit during the Spring Festival | 0.04 | 0.07 | 0.00 | *** |
|  |  | Frequent home visit | 0.00 | 0.00 | 999.00 |  |
|  |  | Children visiting during vacations | 0.01 | 0.01 | 0.00 | *** |
| Class 5 compared to Class 2 | Father-child communication | Frequent phone call | 0.12 | 0.05 | 0.00 | *** |
|  |  | Frequent video call | 0.02 | 0.01 | 0.00 | *** |
|  |  | Home visit during the Spring Festival | 0.79 | 0.41 | 0.61 |  |
|  |  | Frequent home visit | 1.11 | 0.40 | 0.78 |  |
|  |  | Children visiting during vacations | 0.00 | 0.00 | 999.00 |  |
|  | Mother-child communication | Frequent phone call | 0.07 | 0.03 | 0.00 | *** |
|  |  | Frequent video call | 0.00 | 0.00 | 999.00 |  |
|  |  | Home visit during the Spring Festival | 0.44 | 0.27 | 0.04 | * |
|  |  | Frequent home visit | 0.44 | 0.16 | 0.00 | *** |
|  |  | Children visiting during vacations | 0.44 | 0.34 | 0.10 |  |
| Class 5 compared to Class 1 | Father-child communication | Frequent phone call | 0.09 | 0.03 | 0.00 | *** |
|  |  | Frequent video call | 0.00 | 0.00 | 999.00 |  |
|  |  | Home visit during the Spring Festival | 0.20 | 0.10 | 0.00 | *** |
|  |  | Frequent home visit | 0.58 | 0.14 | 0.00 | *** |
|  |  | Children visiting during vacations | 0.00 | 0.00 | 999.00 |  |
|  | Mother-child communication | Frequent phone call | 0.06 | 0.02 | 0.00 | *** |
|  |  | Frequent video call | 0.00 | 0.00 | 999.00 |  |
|  |  | Home visit during the Spring Festival | 0.19 | 0.08 | 0.00 | *** |
|  |  | Frequent home visit | 0.40 | 0.10 | 0.00 | *** |
|  |  | Children visiting during vacations | 0.00 | 0.00 | 999.00 |  |
| Class 4 compared to Class 3 | Father-child communication | Frequent phone call | 0.53 | 0.15 | 0.00 | *** |
|  |  | Frequent video call | 1.01 | 0.59 | 0.99 |  |
|  |  | Home visit during the Spring Festival | 0.22 | 0.21 | 0.00 | *** |
|  |  | Frequent home visit | 0.01 | 0.02 | 0.00 | *** |
|  |  | Children visiting during vacations | 1.99 | 1.18 | 0.40 |  |
|  | Mother-child communication | Frequent phone call | 0.53 | 0.11 | 0.00 | *** |
|  |  | Frequent video call | 0.58 | 0.16 | 0.01 | * |
|  |  | Home visit during the Spring Festival | 0.11 | 0.17 | 0.00 | *** |
|  |  | Frequent home visit | 0.00 | 0.00 | 999.00 |  |
|  |  | Children visiting during vacations | 0.00 | 0.00 | 999.00 |  |
| Class 4 compared to Class 2 | Father-child communication | Frequent phone call | 0.14 | 0.05 | 0.00 | *** |
|  |  | Frequent video call | 0.02 | 0.01 | 0.00 | *** |
|  |  | Home visit during the Spring Festival | 1.86 | 0.93 | 0.36 |  |
|  |  | Frequent home visit | 0.20 | 0.07 | 0.00 | *** |
|  |  | Children visiting during vacations | 0.00 | 0.00 | 999.00 |  |
|  | Mother-child communication | Frequent phone call | 0.13 | 0.05 | 0.00 | *** |
|  |  | Frequent video call | 0.00 | 0.00 | 999.00 |  |
|  |  | Home visit during the Spring Festival | 1.16 | 0.69 | 0.82 |  |
|  |  | Frequent home visit | 0.07 | 0.08 | 0.00 | *** |
|  |  | Children visiting during vacations | 0.00 | 0.00 | 999.00 |  |
| Class 4 compared to Class 1 | Father-child communication | Frequent phone call | 0.10 | 0.03 | 0.00 | *** |
|  |  | Frequent video call | 0.00 | 0.00 | 999.00 |  |
|  |  | Home visit during the Spring Festival | 0.48 | 0.25 | 0.03 | * |
|  |  | Frequent home visit | 0.10 | 0.03 | 0.00 | *** |
|  |  | Children visiting during vacations | 0.00 | 0.00 | 999.00 |  |
|  | Mother-child communication | Frequent phone call | 0.12 | 0.03 | 0.00 | *** |
|  |  | Frequent video call | 0.00 | 0.00 | 999.00 |  |
|  |  | Home visit during the Spring Festival | 0.50 | 0.24 | 0.04 | * |
|  |  | Frequent home visit | 0.06 | 0.07 | 0.00 | *** |
|  |  | Children visiting during vacations | 51423.71 | 0.00 | 999.00 |  |
| Class 3 compared to Class 1 | Father-child communication | Frequent phone call | 0.19 | 0.06 | 0.00 | *** |
|  |  | Frequent video call | 0.00 | 0.00 | 999.00 |  |
|  |  | Home visit during the Spring Festival | 2.19 | 2.24 | 0.60 |  |
|  |  | Frequent home visit | 9.35 | 15.90 | 0.60 |  |
|  |  | Children visiting during vacations | 0.00 | 0.00 | 999.00 |  |
|  | Mother-child communication | Frequent phone call | 0.23 | 0.07 | 0.00 | *** |
|  |  | Frequent video call | 0.00 | 0.00 | 999.00 |  |
|  |  | Home visit during the Spring Festival | 4.48 | 7.05 | 0.62 |  |
|  |  | Frequent home visit | 0.00 | 0.00 | 999.00 |  |
|  |  | Children visiting during vacations | 0.42 | 0.36 | 0.10 |  |
| Class 2 compared to Class 3 | Father-child communication | Frequent phone call | 3.75 | 1.52 | 0.07 |  |
|  |  | Frequent video call | 67.41 | 42.93 | 0.12 |  |
|  |  | Home visit during the Spring Festival | 0.12 | 0.12 | 0.00 | *** |
|  |  | Frequent home visit | 0.06 | 0.10 | 0.00 | *** |
|  |  | Children visiting during vacations | 0.00 | 0.00 | 999.00 |  |
|  | Mother-child communication | Frequent phone call | 4.13 | 1.58 | 0.05 |  |
|  |  | Frequent video call | 0.00 | 0.00 | 999.00 |  |
|  |  | Home visit during the Spring Festival | 0.10 | 0.15 | 0.00 | *** |
|  |  | Frequent home visit | 0.00 | 0.00 | 999.00 |  |
|  |  | Children visiting during vacations | 0.01 | 0.01 | 0.00 | *** |
| Class 2 compared to Class 1 | Father-child communication | Frequent phone call | 0.72 | 0.29 | 0.32 |  |
|  |  | Frequent video call | 0.00 | 0.00 | 999.00 |  |
|  |  | Home visit during the Spring Festival | 0.26 | 0.15 | 0.00 | *** |
|  |  | Frequent home visit | 0.52 | 0.17 | 0.01 | * |
|  |  | Children visiting during vacations | 0.00 | 0.00 | 999.00 |  |
|  | Mother-child communication | Frequent phone call | 0.94 | 0.38 | 0.86 |  |
|  |  | Frequent video call | 1.00 | 0.00 | 999.00 |  |
|  |  | Home visit during the Spring Festival | 0.44 | 0.28 | 0.05 |  |
|  |  | Frequent home visit | 0.90 | 0.29 | 0.73 |  |
|  |  | Children visiting during vacations | 0.01 | 0.00 | 0.00 | *** |

*Note:* *p<0.05; **p<0.01; ***p<0.001.

Appendix 3. QQ-plots# for checking the normality of continuous outcome variables


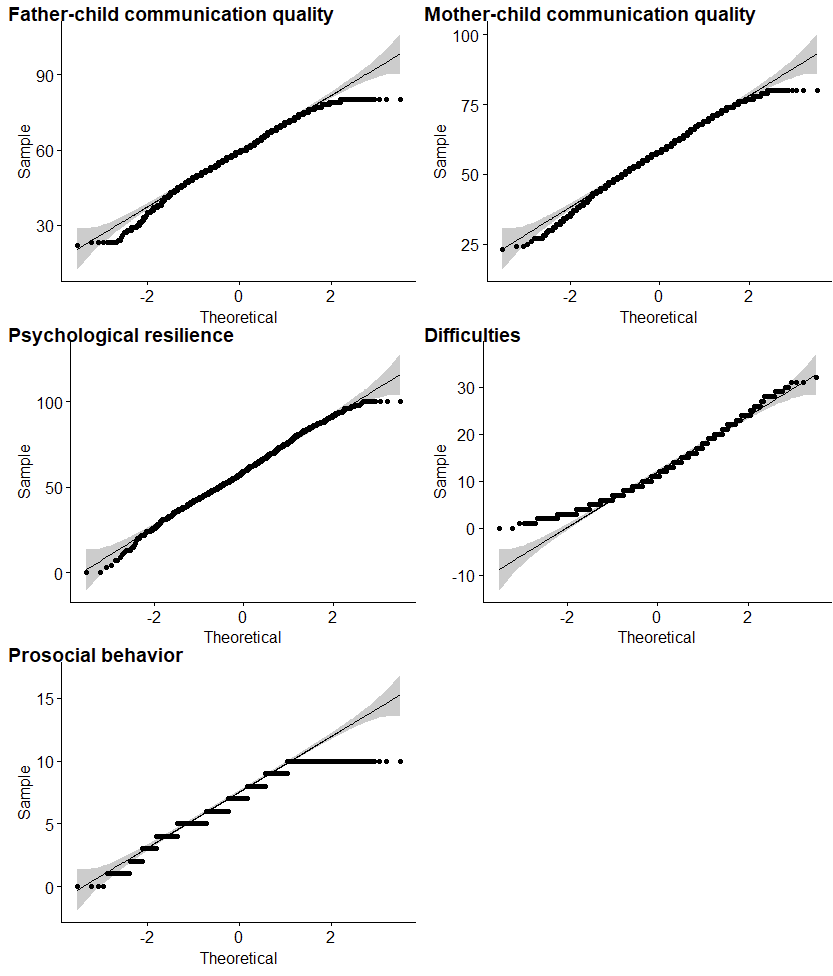


**Concept**: The Q-Q plot compares the quantiles of a dataset with the quantiles of a theoretical distribution. In normality checks, the theoretical distribution used is the normal distribution.

**Plot Construction**: On a Q-Q plot, the x-axis typically represents the quantiles of the theoretical normal distribution, while the y-axis represents the quantiles of the dataset being tested. Each point on the plot corresponds to a specific quantile value in the dataset mapped against the corresponding quantile value in the normal distribution.

**Interpreting the Plot**: If the data are normally distributed, the points on the Q-Q plot will approximately lie on a straight line.

**Appendix 4. Standardized effect sizes for the overall group differences**

| **Comparison** | **Outcome of ANCOVA/Chi-square** | **Omega Squared*** | **Cramer’s V**** | **Interpretation** |
| --- | --- | --- | --- | --- |
| Among latent classes | Father-child communication | 0.06 | - | Medium |
|  | Mother-child communication | 0.06 | - | Medium |
|  | Resilience | 0.06 | - | Medium |
|  | Difficulty | 0.01 | - | Small |
|  | Pro-social behavior | 0.04 | - | Small |
|  | NSSI | - | 0.10 | Small |
|  | Suicidal ideation | - | 0.09 | Small |
| Between never-LBC and latent classes | Father-child communication | 0.07 | - | Medium |
|  | Mother-child communication | 0.06 | - | Medium |
|  | Resilience | 0.07 | - | Medium |
|  | Difficulty | 0.04 | - | Small |
|  | Pro-social behavior | 0.04 | - | Small |
|  | NSSI | - | 0.09 | Small |
|  | Suicidal ideation | - | 0.07 | Small |

*Note:* * Very Small Effect: <0.01; Small Effect: < 0.06; Medium Effect: < 0.14; Large Effect: ≥ 0.14.

** No or very weak: < 0.05; Weak: ≤ 0.10; Moderate: ≤ 0.15; Strong: ≤ 0.25; Very strong: >0.25.

Appendix 5. Sensitivity analysis #1 (excluding parental home visit during the Spring Festival as indicator variable)

**Table S1. LCA model fit index**

| Model | AIC | BIC | aBIC | Entropy | Class proportions |
| --- | --- | --- | --- | --- | --- |
| 2-class | 9517.85 | 9601.70 | 9547.71 | 0.86 | 0.65/0.35 |
| 3-class | 9169.84 | 9298.09 | 9215.51 | 0.91 | 0.15/0.32/0.53 |
| 4-class | 8887.83 | 9060.47 | 8949.31 | 0.88 | 0.19/0.37//0.15/0.29 |
| 5-class | 8741.32 | 8958.35 | 8818.60 | 0.91 | 0.37/0.24/0.19/0.07/0.13 |
| 6-class | 8629.52 | 8890.94 | 8722.61 | 0.90 | 0.20/0.33/0.05/0.13/0.15/0.14 |

**Figure S1. Class probability based on parent-child communication**

**Table S2. The associations between sociodemographic factors and class membership**

| **Reference group: Class 5 (n=139)** | **Class 1 (n=241)** | | | **Class 2 (n=69)** | | | **Class 3 (n=197)** | | | **Class 4 (n=379)** | | |
| --- | --- | --- | --- | --- | --- | --- | --- | --- | --- | --- | --- | --- |
|  | β | OR | 95% CI | β | OR | 95% CI | β | OR | 95% CI | β | OR | 95% CI |
| Age | -0.23* | 0.79 | [-0.41, -0.05] | -0.21 | 0.81 | [-0.45, 0.03] | -0.22* | 0.81 | [-0.40, -0.03] | -0.03 | 0.97 | [-0.19, 0.13] |
| Gender: Female (ref: male) | 0.16 | 1.17 | [-0.29, 0.61] | 0.04 | 1.04 | [-0.58, 0.65] | 0.05 | 1.05 | [-0.41, 0.51] | -0.19 | 0.83 | [-0.60, 0.21] |
| Residency: rural (ref: urban) | -0.46 | 0.63 | [-0.94, 0.03] | -1.43*** | 0.24 | [-2.07, -0.80] | 0.07 | 1.07 | [-0.45, 0.59] | -0.17 | 0.84 | [-0.62, 0.28] |
| Father's education (ref: Middle school and below) |  |  |  |  |  |  |  |  |  |  |  |  |
| College and above | 1.30 | 3.67 | [-0.47, 3.07] | 1.24 | 3.47 | [-0.73, 3.22] | 0.94 | 2.57 | [-0.94, 2.83] | 0.03 | 1.03 | [-1.78, 1.84] |
| High school or secondary technical school | -0.33 | 0.72 | [-1.01, 0.35] | 0.17 | 1.19 | [-0.68, 1.03] | -0.44 | 0.64 | [-1.16, 0.27] | -0.49 | 0.61 | [-1.13, 0.15] |
| Mother's education (ref: Middle school and below) |  |  |  |  |  |  |  |  |  |  |  |  |
| College and above | -1.29 | 0.28 | [-2.69, 0.11] | -0.26 | 0.77 | [-1.85, 1.33] | -1.33 | 0.26 | [-2.85, 0.18] | -0.62 | 0.54 | [-1.88, 0.64] |
| High school or secondary technical school | 0.10 | 1.10 | [-0.71, 0.90] | 0.14 | 1.15 | [-0.89, 1.16] | -0.32 | 0.73 | [-1.20, 0.57] | -0.13 | 0.88 | [-0.91, 0.65] |
| Only child (ref: no) | 0.12 | 1.13 | [-0.35, 0.59] | -0.20 | 0.82 | [-0.88, 0.47] | 0.17 | 1.18 | [-0.31, 0.65] | -0.16 | 0.85 | [-0.59, 0.27] |
| Perceived economic status (ref: poor) |  |  |  |  |  |  |  |  |  |  |  |  |
| Fair | 0.24 | 1.27 | [-0.66, 1.14] | -0.15 | 0.86 | [-1.29, 1.00] | 1.26* | 3.53 | [0.09, 2.44] | 0.04 | 1.04 | [-0.66, 0.75] |
| Wealthy | 0.90 | 2.47 | [-0.09, 1.89] | 0.43 | 1.54 | [-0.82, 1.69] | 1.46* | 4.30 | [0.20, 2.72] | 0.19 | 1.21 | [-0.64, 1.01] |
| Availability of electronic devices and vehicles | 0.53*** | 1.70 | [0.36, 0.70] | 0.22 | 1.25 | [-0.01, 0.45] | 0.47*** | 1.59 | [0.29, 0.64] | 0.10 | 1.10 | [-0.05, 0.24] |
| Number of friends | 0.05 | 1.05 | [0.00, 0.10] | 0.04 | 1.04 | [-0.01, 0.09] | 0.04 | 1.04 | [-0.01, 0.09] | 0.05* | 1.05 | [0.00, 0.10] |

*Note:* OR=odds ratio; *p<0.05; **p<0.01; ***p<0.001.

**Table S3. Differences among the latent classes of LBC and between latent classes and never-LBC**

| **Variable** | **Class 1 (n=241)** | | **Class 2 (n=69)** | | **Class 3 (n=197)** | **Class 4 (n=379)** | **Class 5 (n=139)** | **ANCOVA or χ2 tests among latent classes of LBC** | | **Never-LBC (n=1158)** | **ANCOVA or χ2 tests between latent classes and never-LBC** | |  |
| --- | --- | --- | --- | --- | --- | --- | --- | --- | --- | --- | --- | --- | --- |
|  |  |  |  |  |  |  |  | Test statistics | Post hoc analysis# |  | Test statistics | Post hoc analysis## |  |
|  |  |  |  |  |  |  |  |  |  |  |  |  |  |
| Father-child communication | | 61.25  (10.26) | | 58.71  (10.15) | 58.39  (10.48) | 55.88  (11.00) | 55.27  (10.56) | 7.23*** | 1>4; 1>5 | 58.55  (10.37) | 5.56*** | never-LBC<1 |  |
| Mother-child communication | | 60.76  (10.04) | | 59.65  (9.25) | 56.78  (10.01) | 56.42  (9.97) | 54.55  (10.24) | 6.94*** | 1>3; 1>4; 1>5 | 60.07  (11.08) | 6.41*** | never-LBC<1 |  |
| Psychological resilience | | 63.76  (16.32) | | 56.55  (18.08) | 56.77  (15.96) | 55.54  (15.84) | 53.96  (15.55) | 6.63*** | 1>2; 1>3; 1>4; 1>5 | 57.56  (16.45) | 15.31*** | never-LBC>5 |  |
| Difficulties | | 11.66  (5.43) | | 12.13  (5.42) | 12.80  (5.66) | 12.96  (5.38) | 13.53  (5.44) | 2.61* | 1<5 | 12.65  (5.48) | 7.52*** | never-LBC<3; never-LBC<4; never-LBC<5; |  |
| Prosocial behavior | | 7.58  (1.92) | | 7.07  (2.21) | 6.83  (2.00) | 6.77  (2.02) | 6.43  (1.85) | 4.71*** | 1>3; 1>4; 1>5 | 6.95  (2.01) | 8.71*** | never-LBC<1 |  |
| Nonsuicidal self-injury | | 25 (10.4%) | | 7  (10.1%) | 35  (17.8%) | 68  (17.9%) | 23  (16.5%) | 9.00 | - | 158  (15.4%) | 17.75** | never-LBC<3; never-LBC<4 |  |
| Suicidal ideation | | 46  (19.1%) | | 21  (30.4%) | 53  (26.9%) | 108  (28.5%) | 37  (26.6%) | 8.05 | - | 265  (25.9%) | 12.04* | never-LBC<4 |  |

*Note:* LBC=left-behind children; Adjustment for children’s age, gender, residency, parental education level, only child status, household income status, and number of friends;

# Tukey's tests were used for continuous variables and chi-square independence test was used for categorical variables;

## Dunnett's test were used for continuous variables and chi-square independence test was used for categorical variables; *p<0.05; **p<0.01; ***p<0.001.
